# Necroptosis Mediates TNF-Induced Toxicity of Hippocampal Neurons

**DOI:** 10.1155/2014/290182

**Published:** 2014-07-01

**Authors:** Shan Liu, Xing Wang, Yun Li, Lei Xu, Xiaoliang Yu, Lin Ge, Jun Li, Yongjin Zhu, Sudan He

**Affiliations:** ^1^Cyrus Tang Hematology Center, Jiangsu Institute of Hematology, First Affiliated Hospital, Soochow University, 199 Ren Ai Road, Suzhou Industrial Park, Suzhou, Jiangsu 215123, China; ^2^Department of Physiology and Neurobiology, School of Biology & Basic Medical Science, Soochow University, Suzhou 215123, China

## Abstract

Tumor necrosis factor-*α* (TNF-*α*) is a critical proinflammatory cytokine regulating neuroinflammation. Elevated levels of TNF-*α* have been associated with various neurodegenerative diseases such as Alzheimer's disease and Parkinson's disease. However, the signaling events that lead to TNF-*α*-initiated neurotoxicity are still unclear. Here, we report that RIP3-mediated necroptosis, a form of regulated necrosis, is activated in the mouse hippocampus after intracerebroventricular injection of TNF-*α*. RIP3 deficiency attenuates TNF-*α*-initiated loss of hippocampal neurons. Furthermore, we characterized the molecular mechanism of TNF-*α*-induced neurotoxicity in HT-22 hippocampal neuronal cells. HT-22 cells are sensitive to TNF-*α* only upon caspase blockage and subsequently undergo necrosis. The cell death is suppressed by knockdown of CYLD or RIP1 or RIP3 or MLKL, suggesting that this necrosis is necroptosis and mediated by CYLD-RIP1-RIP3-MLKL signaling pathway. TNF-*α*-induced necroptosis of HT-22 cells is largely independent of both ROS accumulation and calcium influx although these events have been shown to be critical for necroptosis in certain cell lines. Taken together, these data not only provide the first *in vivo* evidence for a role of RIP3 in TNF-*α*-induced toxicity of hippocampal neurons, but also demonstrate that TNF-*α* promotes CYLD-RIP1-RIP3-MLKL-mediated necroptosis of hippocampal neurons largely bypassing ROS accumulation and calcium influx.

## 1. Introduction

Massive loss of a particular subset of neurons is a pathological hallmark of neurodegenerative diseases such as Alzheimer's disease (AD), Parkinson's disease (PD), and multiple sclerosis (MS). Cytokine-driven neuroinflammation and neurotoxicity have been implicated in the initiation and progression of these devastating diseases [1]. Ample evidence suggests that tumor necrosis factor-*α* (TNF-*α*) is a key proinflammatory cytokine regulating neuroinflammation and plays roles in both homeostasis and disease pathophysiology in the central nervous system (CNS) [2]. TNF-*α* is commonly elevated in the clinic and animal models of neurodegenerative diseases. For example, increased level of TNF-*α* is detected in the brain and plasma in AD patients and mouse models of AD. In CNS, TNF-*α* is mainly produced by activated microglia and astrocytes in response to various stimuli including infection and injury. Genetic deletion of TNFR1 has been shown to attenuate the production of the amyloid-*β*  (A*β*) and to improve impairments in mice with AD [3, 4]. Moreover, deficiency of TNF-*α* or TNF receptor protects against dopaminergic neurotoxicity [5, 6]. Therefore, overproduction of TNF-*α* is strongly linked with neuronal damage, and blockage of TNF-*α*-mediated neurotoxic pathway emerges as an attractive strategy for the treatment of degenerative diseases such as AD and PD. Although TNF-*α* has been shown to be neurotoxic to cultured neurons by promoting glutamate production [7], the signaling events that lead to TNF-*α*-initiated neurotoxicity are not yet understood.

As a pleiotropic factor, TNF-*α* is involved in diverse cellular responses including apoptosis and necrosis. TNF family of cytokines, such as TNF-*α*, TRAIL, and FasL, triggers apoptosis by recruiting and activating caspase-8 through the adaptor protein FADD. In some cell types, suppression of caspase-8 or FADD sensitizes cells to programmed necrosis (termed necroptosis) in response to these cytokines as well as ligands of Toll-like receptors (TLRs) [8, 9]. Necroptosis depends on the formation of a necrosome complex, which contains receptor-interacting kinase-1 (RIP1) [10], receptor-interacting kinase-3 (RIP3) [11–13], and mixed lineage kinase domain-like protein (MLKL) [14, 15]. In TNF-*α*-induced necroptosis, deubiquitination of RIP1 by cylindromatosis (CYLD) is a critical process for necrosome formation and activation [16, 17]. Although downstream mechanisms mediating execution of necroptosis remain to be elucidated, reactive oxygen species (ROS) accumulation [13, 18] and calcium influx [19] have been shown to be critical for necroptosis in certain cell lines.

The connection between necroptosis and neuronal damage has been suggested by studies demonstrating a protective effect of necroptosis inhibitor on brain injury in experimental stroke and trauma models [20, 21]. We therefore hypothesize that necroptosis is activated during neuroinflammation and further drives neurotoxicity. To this end, we used RIP3-deficient mice to determine the regulation of necroptosis in TNF-*α*-induced neurotoxicity* in vivo*. Here, we demonstrated that deficiency of RIP3 alleviates the loss of hippocampal neurons in the mouse hippocampus after intracerebroventricular injection of TNF-*α*. Using an* in vitro* hippocampal neuronal model, we provided a detailed molecular characterization of TNF-*α*-induced death of hippocampal neurons.

## 2. Materials and Methods

### 2.1. Animal Models

RIP3 knockout mice were generated as described previously [11] and crossed to C57BL/6 mice for ten generations. Female wild-type and RIP3 knockout mice at 6–8 weeks of age received intracerebroventricular injection of TNF-*α*. In brief, 2.5 *μ*g or 5 *μ*g TNF-*α* was dissolved in PBS to make a total volume of 20 *μ*L and then injected into each lateral ventricle. The control group mice received 20 *μ*L PBS. After 3 days, mice were scarified and the proteins were extracted from hippocampus and subjected to western blot analysis. Morphology of hippocampal neurons was analyzed by Nissl staining of brain sections. All animal experiments were performed in accordance with protocols by the Institutional Animal Care and Use Committee at Soochow University.

### 2.2. Reagents

Dulbecco's modified Eagle's medium (DMEM) was from Thermo. Penicillin/streptomycin, L-Glutamine, and fetal bovine serum (FBS) were from GIBCO. BHA, NAC, phosphate buffered saline (PBS), and Lanthanum(III) chloride heptahydrate (LaCl_3_ *·*7H_2_O) were from Sigma. Recombinant TNF-*α* was purified as described previously [11]. z-VAD was from Bachem. Necrostatin-1 was from Alexis Biochemicals. Propidium Iodide was from Biouniquer. The following antibodies were used for western blotting: mouse RIP3 (Prosci, 2283), RIP1 (BD Biosciences, 610459), mouse CYLD (Cell Signaling, 437700), caspase-3 (Cell Signaling, 9662), and *β*-actin (Sigma).

### 2.3. Cell Culture

Mouse hippocampal neuron (HT-22) cells were a gift from the Lab of Dr. Zhenghong Qin (Soochow University). Mouse embryonic fibroblasts (MEFs) were isolated from day 14.5–15.5 embryos. These cells were grown in DMEM supplemented with 10% fetal bovine serum.

### 2.4. Plasmids and Oligos

Lentiviral expression construct containing mouse RIP3 was amplified from RIP3 plasmid with primers containing an N-terminal Flag epitope and then cloned into pCAG-MCS-IRES vector that was a gift from the Lab of Dr. Yun Zhao (Soochow University). Lentiviral expression construct containing RIP3-RHIM domain mutant (RIP3-RHIM-Mut) or RIP3 kinase mutant (RIP3 K51A) was generated by QuikChange Lightning Site-Directed Mutagenesis Kit (Agilent Technologies). Mouse RIP3, RIP1, MLKL, and CYLD siRNAs were synthesized by GenePharma: RIP3-1 (cccgacgaugucuucugucaa), RIP3-2 (cuccuuaaagucaauaaacau), RIP1-1 (ccacuagucugacugauga), RIP1-2 (ucaccaauguugcaggaua), CYLD-1 (uccauugaggauguaaauaaa), CYLD-2 (aaggguugaaccauuguuaaa), MLKL-1 (gagauccaguucaacgaua), and MLKL-2 (uaccaucaaaguauucaacaa).

### 2.5. Nissl Staining

The mice were sacrificed 72 h after intracerebroventricular injection of TNF*α*. Brains were dissected out of the skull and put in 4% paraformaldehyde to fix the tissue for 24 hours at room temperature and then stored in 30% sucrose phosphate buffer overnight until the tissue sank to the bottom of the solution. 20 *μ*m sections were cut in the coronal plane using a freezing microtome (Leica CM19500) and mounted on gelatin coated slides. The sections were further stained in 0.1% cresyl violet solution (Sigma-Aldrich) at 37°C for several minutes. Rinse quickly in distilled water followed by differentiation in 95% ethyl alcohol and check microscopically for best result. Dehydrate in 100% alcohol and clear in xylene. Finally, the sections were mounted using a neutral balsam and photos were taken under microscope.

### 2.6. Western Blot Analysis

Cell pellets were lysed in lysis buffer containing 20 mM Tris-Hcl (pH 8.0), 150 mM NaCl, 1% Triton X-100, 1% Glycerol, 0.5 mM DTT, 1 mM Na3VO4, 25 mM *β*-glycerol-phosphate, and 1 mM PMSF supplemented with protease inhibitor cocktail (Roche). The mouse tissue was grinded and resuspended in lysis buffer with 0.1% SDS. The resuspended cell pellet or tissue was vortexed for 10 seconds, then incubated on ice for 20 min, and then centrifuged at 20,000 g for 20 min. Protein concentration was determined by Quick Start Bradford 1x Dye Reagent (Bio-Rad). The protein samples were prepared for western blot analysis.

### 2.7. Generation of Stable Cell Lines

293T cells werecotransfected with lentiviral expression construct and packaging plasmids mix, and viral particles were collected after 48 hours and 72 hours. HT-22 cells were infected with lentivirus containing RIP3, RIP3K51A, and RIP3-RHIM-Mut, respectively. 72 hours later cells were selected with GFP by fluorescence-activated cell sorting.

### 2.8. Transfection and Cell Viability Assay

HT-22 cells were transfected with siRNAs by Lipofectamine RNAiMAX Reagent (Invitrogen) for 60 h and then treated with the indicated drug for about 20 h. Cell survival was determined by Cell Titer-Glo Luminescent Cell Viability Assay kit (Promega).

## 3. Results

### 3.1. The Regulation of RIP3 in TNF*-* *α*-Induced Toxicity of Hippocampal Neurons* In Vivo*

**Figure 1:**
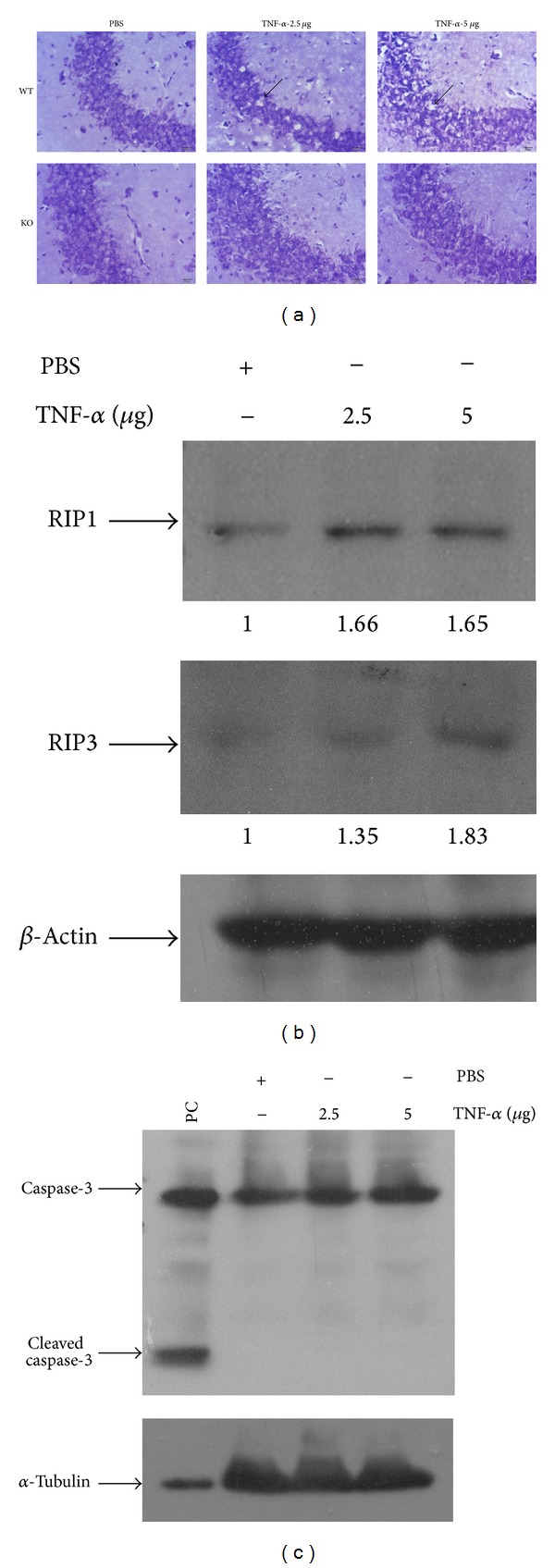
The regulation of RIP3 in TNF-*α*-induced toxicity of hippocampal neurons* in vivo*. (a) Nissl staining of hippocampal neurons 72 h after treatment. Wild-type (WT) and RIP3 knockout (KO) mice received intracerebroventricular injection of PBS or the indicated dose of TNF-*α*. The neurons of brain sections from WT and KO mice were analyzed by Nissl staining (*n* = 7) and morphology of hippocampal CA3 region was shown. Arrows indicate the loss of hippocampal neurons. (b) and (c) Expressions of RIP1, RIP3, and caspase-3 in the hippocampus after TNF*-* *α* treatment. Proteins extracted from hippocampus in the wild-type mice treated with PBS or TNF-*α* were analyzed by western blot using indicated antibodies. PC: MEF cells were treated with staurosporine at 150 nM for 15 hours. The results shown here are representative of five mice.

RIP3 is a key molecule regulating necroptosis induced by TNF family cytokines and ligands of TLR3/4. Elevated expression of RIP3 protein is observed in the damage tissues and correlates with active necroptosis during the pathogenesis of diseases such as acute pancreatitis, retinal detachment, and liver injury [11, 22, 23]. To assess the role of necroptosis in TNF-*α*-induced neurotoxicity, we challenged wild-type and RIP3-deficient mice with intracerebroventricular injection of TNF-*α*. Histological analysis with Nissl staining of neurons was performed to evaluate TNF-*α*-induced damage of neurons. We observed that administration of TNF-*α* to wild-type mice caused a reduction in neuronal density in the hippocampus especially CA3 region in a dose-dependent manner as compared with control-treated mice (Figure 1(a)). Notably, no obvious loss of hippocampal neurons was observed in RIP3-deficient mice after treatment of TNF*-* *α* (Figure 1(a)). Moreover, we noticed that the expression levels of RIP1 and RIP3 were increased in the hippocampus after TNF-*α* treatment (Figure 1(b)), while there is no detectable activation of caspase-3 which is an executioner caspase activated via proteolytic cleavage during apoptosis (Figure 1(c)), indicating that necroptosis but not apoptosis is activated by the injection of TNF-*α*. These results indicate that necroptosis is activated in CNS and contributes to the toxicity of hippocampal neurons in response to TNF-*α*.

### 3.2. HT-22 Hippocampal Neurons Are Committed to Necrosis rather than Apoptosis in Response to TNF-*α*

**Figure 2:**
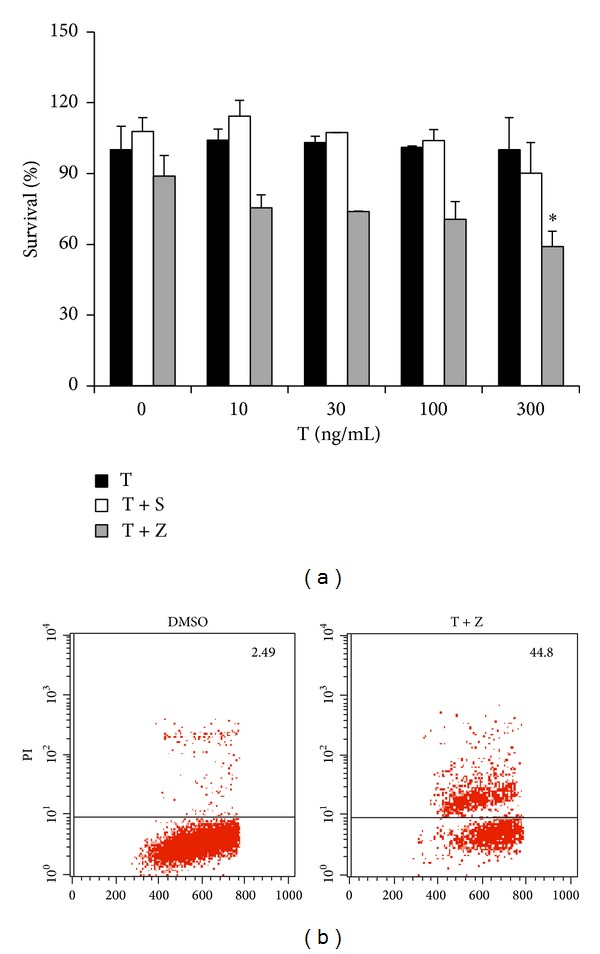
HT-22 hippocampal neurons are committed to necrosis rather than apoptosis in response to TNF-*α*. (a) HT-22 hippocampal neuronal cells were treated as indicated for 20 h. Cell viability was determined by measuring ATP levels. Data are represented as mean ± standard deviation of duplicates. T: TNF-*α*; S: Smac mimetic (100 nM); and Z: z-VAD (20*μ*M). (b) HT-22 hippocampal neurons were treated with DMSO or TNF-*α* (300 ng/mL)/z-VAD for 20 h and then analyzed for PI staining by flow cytometry. Identical concentrations were used in later experiments. Data are represented as mean ± standard deviation of duplicates.  *P < 0.01,  **P < 0.001 versus control. All experiments were repeated three times with similar results.

Having observed RIP3-mediated necroptosis in TNF-*α*-induced toxicity of hippocampal neurons* in vivo*, we sought to clarify the molecule mechanism underling TNF-*α*-induced neurotoxicity in HT-22 hippocampal neuronal cell line, which is often employed as an* in vitro* model of hippocampal neuron. We observed that HT-22 cells were resistant to TNF-*α*, even in the presence of Smac mimetic, a compound which can mimic the function of proapoptotic protein Smac/Diablo and induces apoptosis as a single agent or in combination with TNF-*α* [24, 25] (Figure 2(a)). Notably, addition of caspase inhibitor, z-VAD, sensitized HT-22 cells to death in response to TNF-*α* in a dose-dependent manner (Figure 2(a)). Propidium iodide (PI) positive cells were detected in TNF/z-VAD treated HT-22 cells (Figure 2(b)), suggesting that these cells lost membrane permeability and underwent necrosis. Taken together, these data demonstrate that HT-22 hippocampal neuronal cells are committed to TNF-*α*-induced necrosis rather than apoptosis.

### 3.3. TNF-*α* *-*Induced Necrosis of HT-22 Cells Is Mediated by CYLD-RIP1-RIP3-MLKL Signaling Pathway

**Figure 3:**
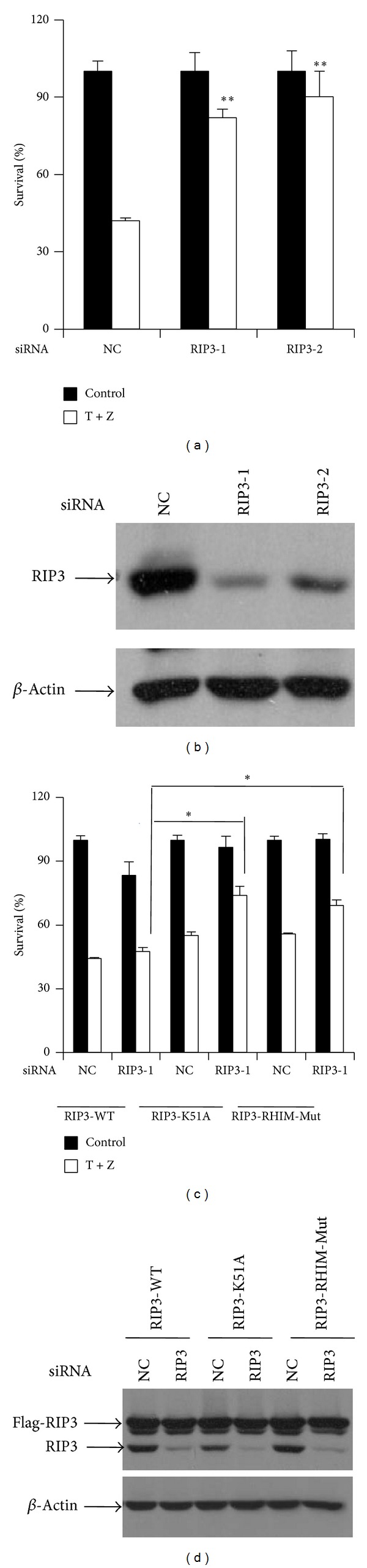
TNF-*α*-induced necrosis of HT-22 cells depends on RIP3 and its kinase activity. (a) HT-22 cells were transfected with the negative control (NC) or RIP3 siRNAs. After 60 h, cells were treated with control or TNF-*α*/z-VAD for another 20 h and then cell viability was determined by measuring ATP levels. Data were represented as mean ± standard deviation of duplicates.  *P < 0.01,  **P < 0.001 versus NC-T + Z. (b) The knockdown efficiency of RIP3 RNAi. Cell lysates were collected 60 h after transfection and subjected to western blot analysis of RIP3 and *β*-actin levels. (c) HT-22 cells stably expressing a siRNA-resistant WT-RIP3 or RIP3-K51A or RIP3-RHIM-Mut were transfected with the control or RIP3 siRNAs. After 60 h, cells were treated with control or TNF-*α*/z-VAD for 20 h and then cell viability was determined by measuring ATP levels. Data were represented as mean ± standard deviation of duplicates.  *P < 0.01,  **P < 0.001 versus NC-T + Z. WT-RIP3: HT-22 cells stably expressing a siRNA-resistant wild-type form of RIP3; RIP3-K51A: HT-22 cells stably expressing a siRNA-resistant RIP3 kinase dead mutant. RIP3-RHIM Mut: HT-22 cells stably expressing a siRNA-resistant RHIM domain mutant form of RIP3. (d) The knockdown efficiency of RIP3 RNAi. Cell lysates were collected 60 h after transfection and subjected to western blot analysis of RIP3 and *β*-actin levels. All experiments were repeated three times with similar results.

RIP3 kinase is a key determinant for necroptosis. RIP3 protein contains an N-terminal serine/threonine kinase domain and a C-terminal RIP homotypic interaction motif (RHIM). The kinase activity and RHIM domain of RIP3 are critical for its function in mediating necroptosis [11]. We examined the role of RIP3 in TNF-*α*-induced necrosis in HT-22 cells by RNAi approach. Knockdown of endogenous RIP3 greatly blocked TNF-*α*-induced necrosis (Figures 3(a) and 3(b)), which was restored by stable expression of a shRNA-resistant wild-type RIP3, but not a shRNA-resistant kinase dead form or RHIM mutant form of RIP3 (Figures 3(c) and 3(d)), indicating that both kinase activity and RHIM domain of RIP3 are crucial for TNF-*α*-induced necrosis of HT-22 cells.

**Figure 4:**
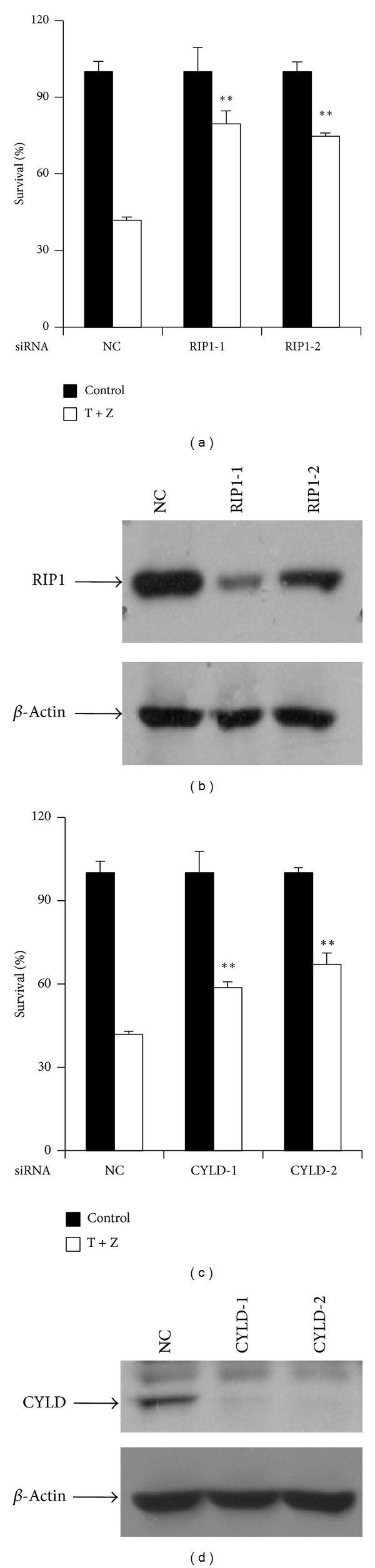
RIP1 and its deubiquitinase CYLD are required for TNF-*α*-induced necrosis of HT-22 cells. (a) HT-22 cells were transfected with the negative control or RIP1 siRNAs. After 60 h, cells were treated with control or TNF-*α*/z-VAD for another 20 h and then cell viability was determined by measuring ATP levels. Data were represented as mean ± standard deviation of duplicates.  *P < 0.01,  **P < 0.001 versus NC-T + Z. (b) The knockdown efficiency of RIP1 RNAi. Cell lysates were collected 60 h after transfection and subjected to western blot analysis of RIP1 and *β*-actin levels. (c) HT-22 cells were transfected with the negative control or CYLD siRNAs. Forty-eight hours after transfection, cells were treated with control or TNF-*α*/z-VAD for another 20 h and then cell viability was determined by measuring ATP levels. Data were represented as mean ± standard deviation of duplicates.  *P < 0.01,  **P < 0.001 versus NC-T + Z. (d) The knockdown efficiency of CYLD RNAi. Cell lysates were collected 60 h after transfection and subjected to western blot analysis of CYLD and *β*-actin levels. All experiments were repeated three times with similar results.

RHIM domain of RIP3 is known to be critical for its interaction with RIP1 during necroptosis [26]. We further tested whether RIP1 is required for TNF-*α*-induced necrosis of HT-22 cells. As shown in Figures 4(a) and 4(b), reducing endogenous RIP1 suppressed TNF-*α*-induced necrosis. In addition, knockdown of CYLD, a deubiquitinase of RIP1, blocked TNF-*α*-induced necrosis of HT-22 cells (Figures 4(c) and 4(d)).

**Figure 5:**
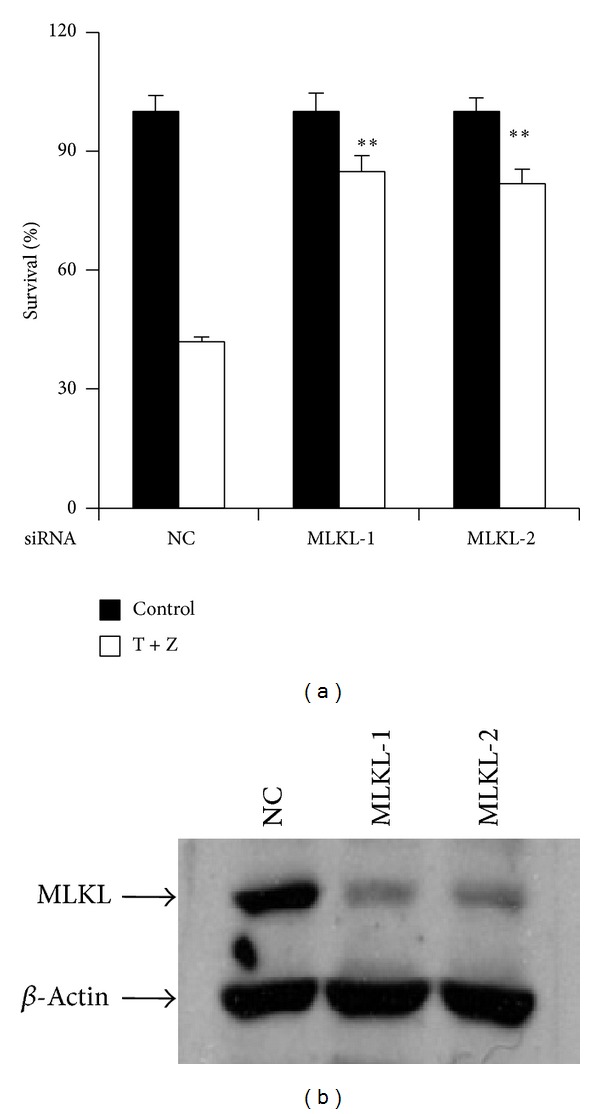
MLKL is essential for TNF-*α*-induced necrosis of HT-22 cells. (a) HT-22 cells were transfected with the negative control or MLKL siRNAs. After 60 h, cells were treated with control or TNF-*α*/z-VAD for another 20 h and then cell viability was determined by measuring ATP levels. Data were represented as mean ± standard deviation of duplicates.  *P < 0.01,  **P < 0.001 versus NC-T + Z. (b) The knockdown efficiency of MLKL RNAi. Cell lysates were collected 60 h after transfection and subjected to western blot analysis of MLKL and *β*-actin levels. All experiments were repeated three times with similar results.

MLKL is a kinase-like protein and functions as a substrate of RIP3. To assess the requirement of MLKL in TNF-*α*-induced necrosis of HT-22 cells, we performed MLKL RNAi in the cells and found knockdown of MLKL efficiently reduced the cell death (Figures 5(a) and 5(b)).

### 3.4. TNF-*α*-Induced Necroptosis of HT-22 Cells Is Largely Independent of ROS Accumulation and Calcium Influx

**Figure 6:**
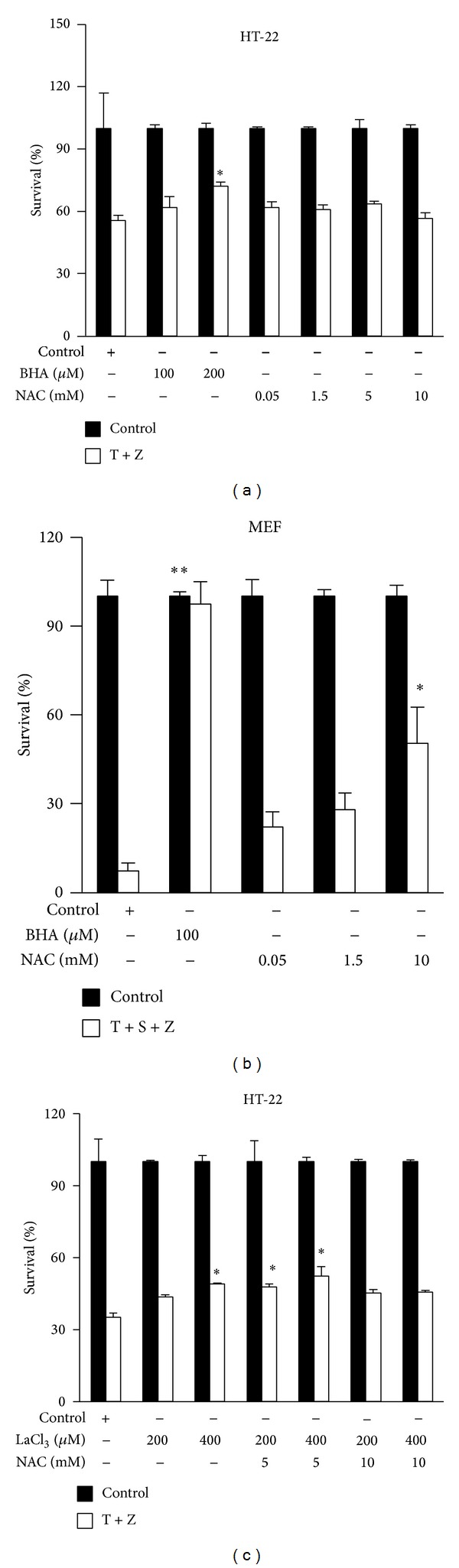
TNF-*α*-induced necrosis of HT-22 cells is largely independent of ROS accumulation and calcium. (a) HT-22 cells were treated with control or BHA or NAC at the indicated concentration 3 h before the treatment of control or TNF-*α*/z-VAD for 20 h; cell viability was determined by measuring ATP levels.  *P < 0.01,  **P < 0.001 versus control-T + Z. (b) MEF cells were treated with control or BMS NAC at the indicated concentration 3 h before the treatment of control or TNF-*α*/Smac mimetic/z-VAD for 20 h; cell viability was determined by measuring ATP levels.  *P < 0.01,  **P < 0.001 versus control-T + S + Z. (c) HT-22 cells were treated with control or LaCl_3_ or NAC at the indicated concentration 3 h before the treatment of control or TNF-*α*/z-VAD for 20 h; cell viability was determined by measuring ATP levels. Data are represented as mean ± standard deviation of duplicates.  *P < 0.01,  **P < 0.001 versus control-T + Z. All experiments were repeated three times with similar results.

We and others have shown that ROS accumulation is required for RIP3-mediated necrosis in certain cell lines such as mouse embryonic fibroblast (MEF) [13, 18], so we evaluated the role of ROS in TNF-*α*-induced necroptosis of HT-22 cells by using two widely used ROS scavengers, butylated hydroxyanisole (BHA) and N-acetylcysteine (NAC). MEF cells are known to undergo necroptosis in response to TNF-*α*, Smac mimetic and z-VAD. In the presence of BHA at 100 *μ*M, the survival rate of HT-22 cells was increased by 13% and around 30% cells still underwent necrosis in response to TNF-*α* plus z-VAD, while TNF-*α*-induced necrosis in MEF cells was entirely prevented by BHA (Figures 6(a) and 6(b)). NAC had no inhibitory effect on TNF-*α*-induced necrosis of HT-22 cells, whereas the survival rate of MEF cells treated with necroptotic stimuli was increased by 40% after the addition of NAC at 10 mM (Figures 6(a) and 6(b)). Recently, calcium influx has been reported to be essential for necroptosis. We tested whether calcium influx is involved in TNF-*α*-induced necroptosis of HT-22. As shown in Figure 6(c), inhibition of calcium influx by the addition of LaCl_3_, a non-voltage-sensitive channel blocker, increased the survival rate of HT-22 by around 14%. Notably, TNF-*α*-induced necroptosis of HT-22 cells largely proceeded even in the presence of both LaCl_3_ and NAC (Figure 6(c)). These results demonstrated that TNF-*α*-initiated necroptosis in HT-22 cells is largely independent of ROS accumulation and calcium influx.

## 4. Discussion 

TNF-*α* is a key mediator of neuroinflammation. Elevated levels of TNF-*α* are associated with various neurodegenerative conditions and contribute to neurotoxicity. The mechanisms underlying TNF-*α*-initiated neurotoxicity are largely unknown. Our present work revealed an important role of necroptosis in TNF-*α*-induced toxicity of hippocampal neurons.

Necroptosis is a form of programmed necrosis which is regulated by RIP1 and RIP3 kinases. Apoptosis is known to negatively regulate necroptosis via active caspase-8, which is able to cleave necrosis regulators including RIP1, RIP3, and CYLD [8]. A recent work has shown that mutating the caspase-8 cleavage site at Asp 215 of CYLD is sufficient to promote necroptosis even in the absence of caspase inhibitor, indicating that caspase-8 prevents necroptosis through processing CYLD [27]. CYLD was originally thought to deubiquitinate RIP1 at the membrane receptor complex and promote the recruitment of RIP1 into the necrosome. Recently, it was shown to control the deubiquitination of RIP1 and it facilitates kinase activation in the necrosome [17]. Among the components of necrosome, MLKL is a functional substrate of RIP3. Upon the phosphorylation of MLKL by RIP3, MLKL forms oligomers and locates to the cell plasma membrane [19, 28, 29]. Although mechanisms of necroptosis are extensively studied, there is little known about the machinery of necroptosis in neuronal cells. In this study, we found that HT-22 hippocampal neurons are committed to necroptosis rather than apoptosis in response to TNF-*α*. Using RNAi approach, we demonstrated that TNF-*α*-induced neuronal necrosis is mediated by CYLD-RIP1-RIP3-MLKL signaling pathway. Our present study suggests that the core components of necrosome have conserved roles in neuronal necrosis initiated by TNF-*α*.

Necroptotic cell death is characterized by disrupted plasma membrane; however, the downstream events of necrosome leading to collapse of membrane are largely unknown. ROS production has been shown to be required for necroptosis in several mouse cell lines including L929, MEF, NIH3T3, and macrophages, whereas it is not involved in necroptosis in HT-29 human colon cancer cells [30]. NADPH oxidase NOX1 and metabolic enzymes have been implicated in the control of ROS production during necroptosis [13, 31]. We have shown that TNF-*α*-induced necroptosis of HT-22 cells mouse hippocampal neurons is largely independent of ROS accumulation. Recently, TRPM7 has been identified to mediate calcium influx through acting downstream of MLKL membrane localization in TNF-*α*-induced necroptosis [28]. In our study, we showed that TNF-*α*-induced necroptosis of HT-22 cells largely proceeds even in the presence of calcium channel inhibitor and ROS scavenger. The data indicate that TNF-*α*-induced necroptosis of HT-22 cells largely bypasses ROS accumulation and calcium influx. Therefore, it is tempting to speculate that different downstream responses activated by necrosome depend on cell type. Future studies are required to clarify crucial downstream events for the execution of necroptosis in hippocampal neurons.

Necroptosis is emerging as an important process involved in various pathological conditions including ischemic injury, acute pancreatitis, inflammatory bowel disease, and neuronal damage. Recent studies have demonstrated that increased level of RIP3 expression in the damage tissue correlates with the induction of necroptosis in the mouse models of acute pancreatitis and inflammatory bowel disease (IBD) [30] and patients with IBD [32]. Interestingly, we observed elevated expressions of RIP3 and RIP1 proteins and neuronal cell death in the hippocampus after intracerebroventricular injection of TNF-*α*. Importantly, genetic deletion of RIP3 reduced the loss of hippocampal neurons after intracerebroventricular injection of TNF-*α*. To our knowledge, the data represent the first* in vivo* evidence for a role of RIP3 in TNF-*α*-induced neurotoxicity of hippocampal neurons. Blockage of necroptosis by necrostatin-1, a chemical inhibitor targeting RIP1 kinase, has provided protective effects on neuronal damage in animal models of brain injury [20], stroke [18], and amyotrophic lateral sclerosis [33]. Our study has demonstrated that necroptosis mediates TNF-*α*-initiated damage of hippocampal neurons. Given the elevated levels of TNF-*α* in the brains during various neurodegenerative diseases, neuronal cells may be susceptible to necroptosis upon stimulation of TNF-*α*, therefore contributing to the pathogenesis of neurodegenerative diseases. Targeted prevention of neuronal necroptosis may provide a novel therapeutic approach for the treatment of the related neurodegenerative diseases.

## 5. Conclusions

Taken together, our study revealed an important role of necroptosis in TNF-*α*-induced neurotoxicity. Necroptosis can be activated in the mouse hippocampus after intracerebroventricular injection of TNF-*α*. RIP3 deficiency attenuates TNF-*α*-initiated loss of hippocampal neurons. HT-22 hippocampal cells are sensitive to TNF-*α* only upon caspase blockage and subsequently undergo necrosis. A detailed molecular characterization demonstrates that TNF-*α*-induced necrosis in HT-22 cells is mediated by CYLD-RIP1-RIP3-MLKL necroptotic signaling pathway and largely independent of both ROS accumulation and calcium influx.
